# Brief History, Preparation Method, and Biological Application of Mesoporous Silica Molecular Sieves: A Narrative Review

**DOI:** 10.3390/molecules28052013

**Published:** 2023-02-21

**Authors:** Qiuping Li, You Zhou

**Affiliations:** 1 School of Pharmacy, Fuzhou Medical College of Nanchang University, Fuzhou 344000, China; 2 Key Laboratory of Chronic Diseases, Fuzhou Medical College of Nanchang University, Fuzhou 344000, China; 3 Technology Innovation Center of Chronic Disease Research in Fuzhou City, Fuzhou Science and Technology Bureau, Fuzhou 344000, China; 4 State Key Laboratory Base of Novel Functional Materials and Preparation Science, School of Materials Science and Chemical Engineering, Ningbo University, Ningbo 315211, China

**Keywords:** mesoporous silica, molecular sieve, synthesis method, development history, biological application

## Abstract

It has been more than 30 years since the first ordered mesoporous silica molecular sieve (MCM-41) was reported, but the enthusiasm for exploiting mesoporous silica is still growing due to its superior properties, such as its controllable morphology, excellent hosting capability, easy functionalization, and good biocompatibility. In this narrative review, the brief history of the discovery of mesoporous silica and several important mesoporous silica families are summarized. The development of mesoporous silica microspheres with nanoscale dimensions, hollow mesoporous silica microspheres, and dendritic mesoporous silica nanospheres is also described. Meanwhile, common synthesis methods for traditional mesoporous silica, mesoporous silica microspheres, and hollow mesoporous silica microspheres are discussed. Then, we introduce the biological applications of mesoporous silica in fields such as drug delivery, bioimaging, and biosensing. We hope this review will help people to understand the history of the development of mesoporous silica molecular sieves and become familiar with their synthesis methods and applications in biology.

## 1. Introduction

Porous materials have been widely used for thousands of years. One of the most well-known materials in this category is activated carbon, which is commonly used as an adsorbent [1,2,3]. Over the past few decades, porous materials, especially nano-scale porous materials, have received widespread attention due to their large surface area, open structure, and so on [4,5,6,7]. Well-established nanoporous materials include mesoporous silica molecular sieves [8], zeolite molecular sieves [9], metal–organic frameworks [10], covalent organic frameworks [11], nanoporous carbon [12], etc. Among these, mesoporous silica molecular sieves have stood out in the field of biological applications because of their adjustable pore size, easy functionalization, high stability, and physiological inertia [13,14,15]. According to the IUPAC nomenclature rules, mesoporous materials usually refer to materials with a pore size between 2 and 50 nm. Moderate-sized apertures ensure that mesoporous silica molecular sieves have a large surface area and are easy to fill with various materials. Additionally, the non-toxic properties of silica have been approved by the FDA (Food and Drug Administration) and EFSA (European Food Safety Authority), and related materials are widely used in the cosmetics and food additive industries [16,17,18,19]. Thus, mesoporous silica molecular sieves have great application potential as a carrier in drug delivery [20], bioimaging [21], biosensing [22], materials, etc., and have become one of the most important materials in the field of biological applications.

## 2. History of Mesoporous Silica Molecular Sieves

The first reported mesoporous silica was discovered by Yanagisawa et al. [23] in 1990. They synthesized an alkyltrimethylammonium–kanemite complex by reacting single-layered polysilicate kanemite with alkyltrimethylammonium chloride solutions, and they found that the SiO_2_ layers in the complexes condensed to form three-dimensional SiO_2_ networks. Two years later (1992), Kresge et al. [24,25] reported a novel ordered self-assembled mesoporous silica molecular sieve prepared by means of a template method, in which the silicate material forms inorganic walls via the hydrolysis polycondensation of tetramethylammonium silicate between ordered surfactant micelles. As they worked in Mobil Corporation Laboratories, they named this mesoporous silica MCM-41 (Mobil Composition of Matter No. 41), and it has been widely circulated since then. Kresge et al. [26,27,28,29] then extended their research to the M41S family of mesoporous molecular sieves, and found that the pore diameter, shape, and connectivity of M41S could be manipulated by controlling the structure and size of surfactant molecules. As scientific breakthroughs always bring booming development in related fields, this surfactant-based template method was soon found to be useful for the synthesis of other ordered mesoporous materials [30,31,32].

Since then, inspired by Kresge’s work, many new families of mesoporous silica materials have been reported by researchers all over the world. For example, to increase silica’s pore size, Zhao et al. [33] (1998) introduced an amphiphilic triblock copolymer with larger molecules as a template agent, and obtained an ordered hexagonal mesoporous silica structure. The resulting mesoporous silica, which was developed at the University of California, Santa Barbara, was denoted as SBA-15, which comes from the abbreviation of “Santa Barbara Amorphous No. 15”. Compared with MCM-41, SBA-15 has the advantages of a larger pore diameter, a thicker pore wall, and better hydrothermal stability, which provide it with wider application prospects. Since its development, the shining SBA family of mesoporous molecular sieves has gradually become one of the hottest research subjects in the field of mesoporous materials [34,35,36,37,38].

Alongside the M41s and SBA families, other important mesoporous silica families include the HMS family [39], FSM family [40], MSU family [41], KIT family [42], FDU family [43], JLU family [44], TUD family [45], COK family [46], HMM family [47], and so on. Key information about the origin of these mesoporous silica families is provided in Table 1. In addition to exploring the synthesis of mesoporous silica, researchers have also conducted significant research on its possible applications.

**Figure 1:**
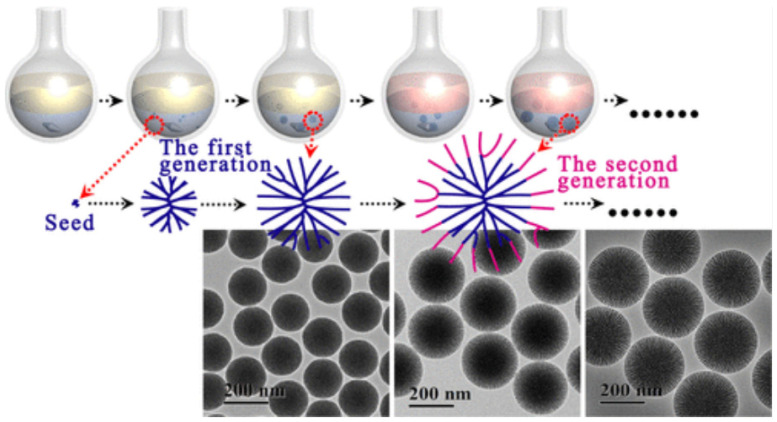
Synthesis process, mechanism of interfacial growth, and representative micrographs of the dendritic mesoporous silica nanospheres. Reproduced with permission from Ref. [57], Copyright 2014, American Chemical Society.

The ideal mesoporous silica for biological applications usually has a moderate pore size (less than 200 nm) and large surface area. With this in mind, researchers have focused their attention on synthesizing mesoporous silica microspheres with nanoscale dimensions. In 1997, Grun et al. [48] reported for the first time the synthesis of submicron spherical MCM-41 particles using a modified Stöber method, which involved adding a cationic surfactant as a template. Before long, Cai et al. [49] obtained spherical MCM-41 particles with an average size of 110 nm by reacting the extremely dilute solution of CTAB with TEOS in a sodium hydroxide medium at 353 K. Kobler et al. [50,51,52] prepared a series of MCM-like near-spherical silica particles with relatively uniform particle sizes between 40 and 150 nm via altering the incorporated functional group and the amount of TEA added in the synthesis. Although mesoporous silica has an abundant number of pores, there are continuous efforts to increase its volume. Some researchers have attempted to add hollow structures to the mesoporous silica to achieve this [53,54,55]. Zhu et al. [56] reported for the first time the synthesis of hollow mesoporous silica spheres via penetrating pore channels across the shells using PVP and CTAB as co-templates, which are uniform in size and have a high drug storage capacity. Another requirement for biological applications is that the material be degradable in vivo. To meet this requirement, Zhao’s group [57] invented a novel monodispersed three-dimensional dendritic mesoporous silica nanosphere that not only had tunable pore size and thickness, but were also biodegradable in simulated body fluid. As shown in Figure 1, the dendritic mesoporous silica nanosphere had a highly uniform particle size and mesostructures, making it a very attractive material for various applications.

## 3. Preparation Methods

As noted earlier, the first ordered mesoporous silica (MCM-41) was synthesized by means of a liquid crystal templating method. Usually, a typical lyotropic liquid crystal template can be prepared by mixing water and surfactant. The synthesis of MCM-41 is based on a silica skeleton precipitated around a lyotropic liquid crystal template formed by mixing quaternary ammonium surfactant with water. In fact, the synthesis of mesoporous silica is usually carried out via a liquid crystal templating method. Template-assisted technology has long been the most utilized method for the synthesis of ordered mesoporous materials, and various surfactant molecules can be used as templates to direct the synthesis of mesoporous silica [58,59,60]. As the liquid crystal template is not a solid template, a method called “soft-matter templating” is often used, and at times the endotemplate method [61]. In general, the structure of the liquid crystal template strongly depends on the conditions in the solution, such as the pH, temperature, and the concentration [62]. 

**Figure 2:**
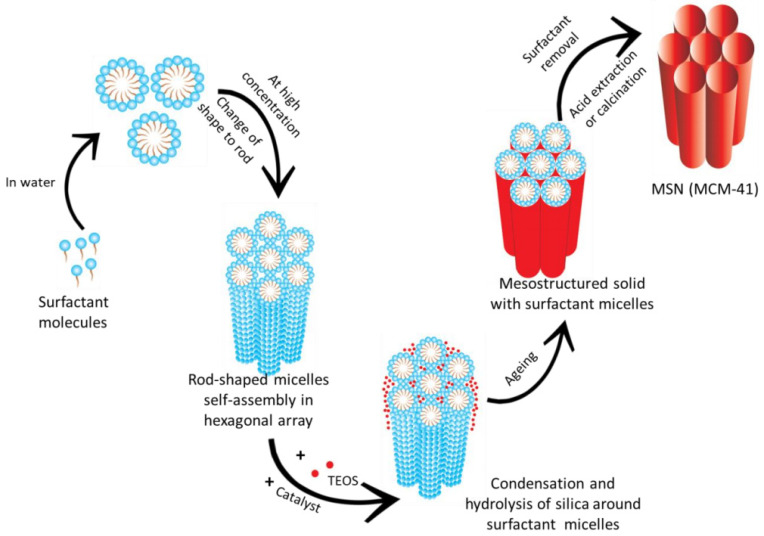
Mechanism of formation of MCM-41. Reproduced with permission from Ref. [63], Copyright 2018, MDPI.

The discovery of MCM-41 promoted research on mesoporous silica materials. Since then, efforts have been made to uncover the exact mechanism behind the formation of mesoporous silica. Narayan et al. [63] clearly demonstrated the formation mechanism of MCM-41 (Figure 2). In addition to the liquid crystal templating mechanism, the synthesis of MCM-41 also involves a sol–gel process wherein the polymerization of silica is carried out around the micelles. Once the silica settles, the porosity can be obtained after the removal of the template from the mesostructured solid via calcination or solvent extraction. Most other types of mesoporous silica have similar synthesis processes. Thus, a typical synthesis process for mesoporous silica can be divided into three steps: (i) choosing an appropriate template and forming a liquid crystal template under certain conditions; (ii) adding the silicon source, waiting for the silica to settle completely around the surfactant micelles, and then performing an aging treatment; and (iii) finally, removing the template.

The original MCM-41 was synthesized in alkaline media and by means of a hydrothermal treatment. Generally, the synthesis is carried out under high temperature (≥100 °C) in an autoclave and requires a long crystallization time. Under certain conditions, the preparation of mesoporous silica can also be performed at room temperature [64,65,66]. In 1994, Huo et al. [67] reported room-temperature synthesis of mesoporous silica under acidic conditions for the first time. Compared to hydrothermal synthesis, the room temperature method is easy to carry out and does not require special equipment, which makes it more suitable for large-scale production. Although the room temperature method has many advantages, the crystallinity of mesoporous silica can be improved by increasing the temperature and pressure of the synthesis process within a certain range. Therefore, although it has been used for decades, the hydrothermal method remains the most efficient technique for the synthesis of mesoporous silica. Additionally, there are continuous efforts to prepare mesoporous silica with the high-temperature hydrothermal method in order to obtain mesoporous silica with unusual stability [68,69,70]. Xiao’s group [71] reported, for the first time, the synthesis of the mesoporous silica JLU-20 in strong acidic media at high temperatures (190 °C) using a mix of fluorocarbon and Pluronic P123 surfactants. Compared with the mesoporous silica prepared with the traditional method, the condensation degree of JLU-20 is higher, indicating a greatly improved thermal stability. Additionally, to further improve the synthetic efficiency, a microwave-assisted technique has been applied to the preparation of mesoporous silica [72,73,74]. It has been demonstrated that the microwave-assisted hydrothermal process has the advantages of a shorter reaction time, lower energy consumption, and more uniform product morphology compared to the conventional autoclave heating method.

**Figure 3:**
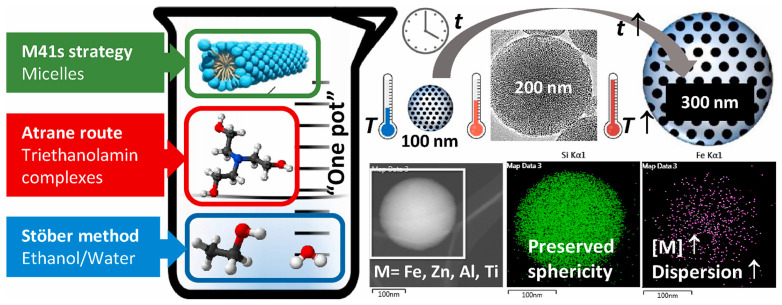
One-pot preparative strategy and representative micrographs of the mesoporous silica functionalized with homogeneous distributed elements (Fe, Zn, Al, or Ti). Reproduced with permission from Ref. [77], Copyright 2022, Elsevier.

Nanoscale mesoporous silica microspheres have attracted great interest because of their great potential for biological applications. The combination of the classical Stöber method and template method has been proven to be a successful strategy for the synthesis of mesoporous silica microspheres [75,76]. The typical Stöber process usually involves the hydrolysis and condensation of tetraethylorthosilicate (TEOS) in an alcohol–water mixture using an ammonia catalyst. In this process, monodisperse spherical silica can be obtained via a seed growth mechanism driven by surface tension. When the surfactant template is present, the particle growth process is accompanied by a cooperative self-assembly process, leading to the formation of spherical mesoporous silica. Recently, Garrido et al. [77] (Figure 3) proposed a reproducible “one-pot” method for the preparation of spherical mesoporous silica that could be functionalized with inorganic species based on a combination of the Atrane and the Stöber methods. This method simultaneously ensures the spherical morphology and dispersed degree of heterogeneous elements, which make it a critical technique for the synthesis of elemental functionalized spherical mesoporous silica.

**Figure 4:**
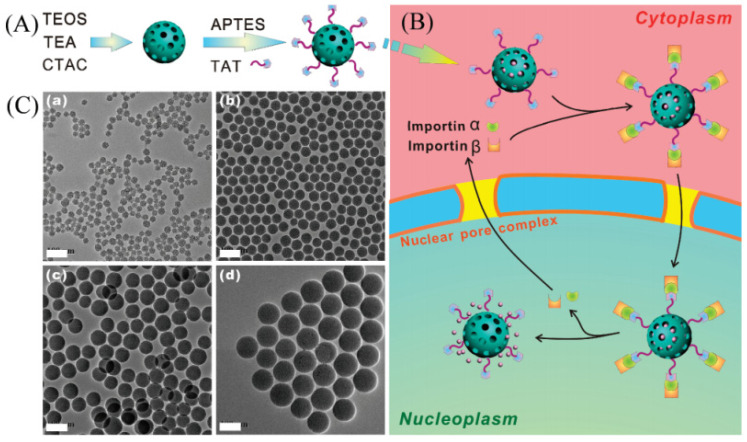
(**A**) Schematic diagram of the procedures for developing mesoporous silica nanoparticles. (**B**) Schematic illustration of transport of DOX-loaded particles across the nuclear membrane. (**C**) TEM images of mesoporous silica nanoparticles with sizes of (**a**) 25, (**b**) 50, (**c**) 67, and (**d**) 105 nm. Scale bars: 100 nm. Reproduced with permission from Ref. [78], Copyright 2012, American Chemical Society.

Furthermore, the seed growth mechanism presented by Stöber has also been extended to prepare mesoporous silica microspheres under different conditions. For example, Pan et al. [78] reported the synthesis of mesoporous silica microspheres with uniform and tunable particle size by using hexadecyl trimethyl ammonium chloride as a template and triethanolamine (TEA) as an alkaline catalyst. The particle size of mesoporous silica microspheres can be tuned by changing the amount of TEA used. As shown in Figure 4, the obtained mesoporous silica microspheres are highly monodispersed and can be used for nuclear-targeted drug delivery.

**Figure 5:**
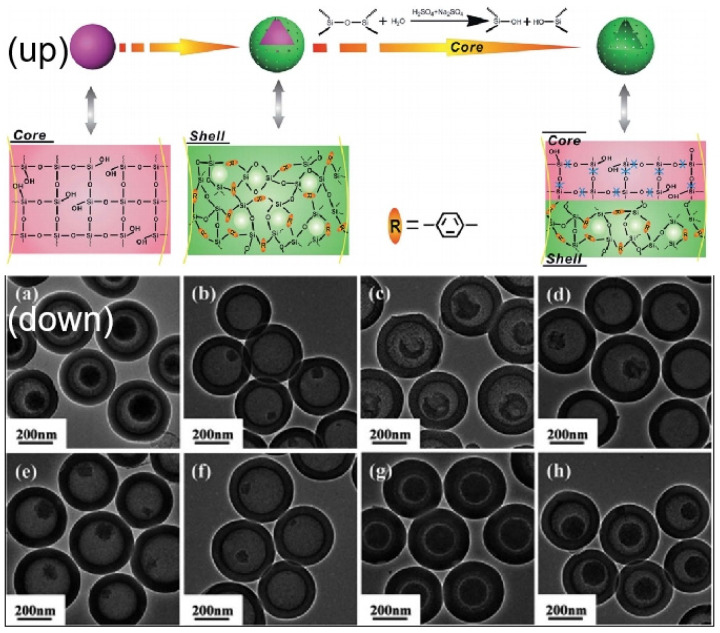
(**Up**) Schematic illustration of the structural difference between the sSiO_2_ core and mSiO_2_ (moSiO_2_) shell, and the synthetic procedure for HMOVs employing the acid etchants (H_2_SO_4_ and Na_2_SO_4_). (**Down**) TEM images of RMOVs converted from sSiO_2_@moSiO_2_ by H_2_SO_4_ plus Na_2_SO_4_ etching for varied hydrothermal time durations. Reproduced with permission from Ref. [86], Copyright 2015, Royal Society of Chemistry.

In order to increase the loading capacity of these materials, efforts have been made to synthesize hollow mesoporous silica microspheres [79]. Soft templating methods such as the micelle templating method [80], microemulsion templating [81], etc., play an important role in the synthesis of hollow mesoporous silica microspheres. Han et al. [82] reported a series of mesoporous silica hollow spheres which were synthesized via a micelle and emulsion dual-templating route. The obtained particles had a diameter in the range of 80~220 nm, constant shell thickness of about 20 nm and constant mesopore size of about 4 nm. In particular, a hard templating method using polymer beads [83], silica colloids [84], or metal oxides [85] as the seed cores was developed to synthesize discrete, uniform, and monodisperse hollow mesoporous silica microspheres to meet the rigorous requirements of biological applications. For example, Wu et al. [86] reported a salt-assisted acid-etching strategy to prepare hollow mesoporous silica (Figure 5). The solid SiO_2_ core templates were prepared first via the classical Stöber process, and then pre-mixed TEOS and octadecyltrimethoxysilane (C_18_TMS) were added to the reaction system to promote the growth of the mesoporous shell around the solid SiO_2_ core. A salt-assisted acid strategy was then used to etch the solid SiO_2_ core of the SiO_2_@mSiO_2_. Further removal of the surfactant C_18_TMS provides highly uniform hollow mesoporous silica nanovehicles (HMSVs). With a similar process, and the addition of 1,4-bis(triethoxysily) benzene, an organosilica-functionalized SiO_2_@moSiO_2_ and HMOVs were subsequently obtained. The as-synthesized HMOVs were found to have high cargo-loading capacity and pH-responsive drug-releasing behavior due to their hollow structure and phenylene-bridged framework. This seed growth and core-etching method based on a colloidal solution of hard templates has been proven to be an effective strategy for the synthesis of hollow mesoporous silica microspheres by many research works in the past decade.

There are also many other novel approaches that have been proposed for the direct generation of mesoporous silica, such as the aerosol-based procedure [87], the biphase stratification approach [88,89,90], the evaporation-induced aggregating assembly method [91,92], and so on.

## 4. Biological Applications

Due to the chemical stability of SiO_2_ matrices, the abundant pores in mesoporous silica are an ideal vessel for a variety of molecules. Furthermore, the excellent biocompatibility of SiO_2_ matrices further broadens mesoporous silica’s application prospects in fields such as drug delivery, bioimaging, biosensing, and so on [93,94,95,96,97,98,99]. 

**Figure 6:**
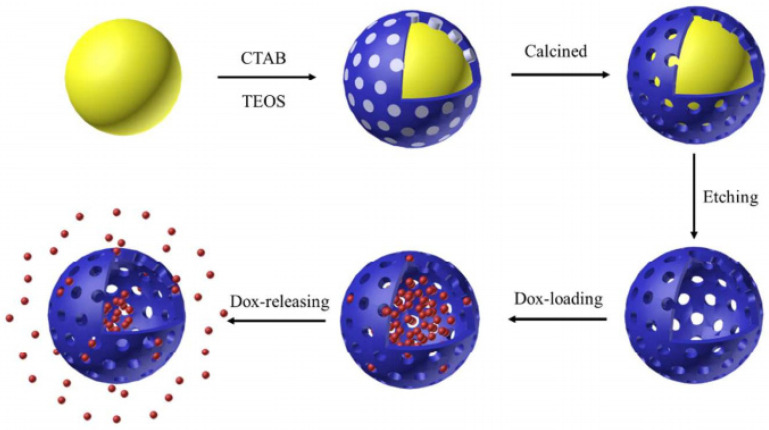
Schematic illustration of the preparation of hollow mesoporous silica used for drug loading and drug releasing. Reproduced with permission from Ref. [105], Copyright 2017, American Chemical Society.

Vallet-Regi et al. [100] were pioneers in using mesoporous silica as a drug carrier. They firstly integrated ibuprofen into two MCM-41 materials with different pore sizes and subsequently examined the in vitro process of releasing the drug to a simulated body fluid. Since then, mesoporous silica has been widely used for drug delivery because of its high drug loading capacity, biocompatibility, and so on. Hollow mesoporous silica microspheres with a uniform particle size have become the most popular drug carrier [101,102,103,104]. Li et al. [105] (Figure 6) prepared mesoporous-silica-coated Au nanoparticles by employing gold nanoparticles (Au) as a core template and surfactant cetyltrimethyl ammonium bromide (CTAB) as the template directing mesoporous structure. After the CTAB and Au templates were removed via calcination and subsequently etching, hollow mesoporous silica nanoparticles were obtained and used to load doxorubicin hydrochloride (DOX), leading to a nanocapsule HMSN (Dox). It has been proven that the drug-releasing behavior of HMSNs (Dox) is consistent with the Fickian diffusion mechanism and can be tuned by adjusting the thickness of the mesoporous shell. 

**Figure 7:**
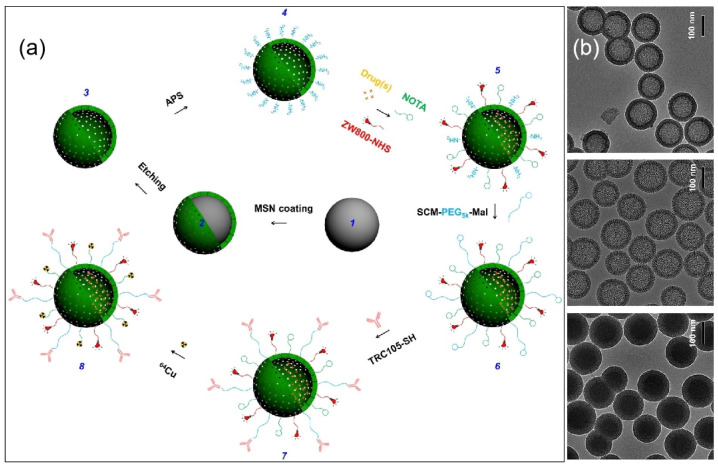
(**a**) Scheme of surface engineering hollow mesoporous silica nanoparticles; (**b**) TEM images of the corresponding nanoparticles. Reproduced with permission from Ref. [109], Copyright 2014, Springer Nature.

To meet the ever-increasing demand for precise treatment, drug delivery systems that can target diseased tissues are urgently needed [106,107,108]. For the treatment of cancer, in particular, a targeted controlled-release drug carrier can lower dosage, prolong drug duration, and prevent damage to healthy tissue. Chen et al. [109] reported multi-functional hollow mesoporous silica nanoparticles with enhanced drug delivery and in vivo imaging properties. As shown in Figure 7, the drug deliverer was prepared using a generally applicable surface engineering technique, and it had a uniform particle size. By grafting a CD105-targeting antibody on the surface of the particles, a 3-fold tumor uptake on average was achieved in 4T1 murine breast-tumor-bearing mice compared with the non-targeted group, as observed by a quantitative PET imaging study via chelating radioactive ^64^Cu to the particles and an in vivo NIRF imaging study via anchoring NIR dye on the particles. Furthermore, its hollow core confers on the drug carrier a 3~15-times-higher DOX loading capacity than that of carriers without a hollow structure, and its highest loading capacity was estimated to be 1129.2 mg/g. Their work showed that a diseased-tissue-targeted drug delivery system can be developed via the elaborated surface functionalization of mesoporous silica, suggesting that in vivo targeted cancer therapy can benefit from advances in mesoporous silica nanotechnology.

**Figure 8:**
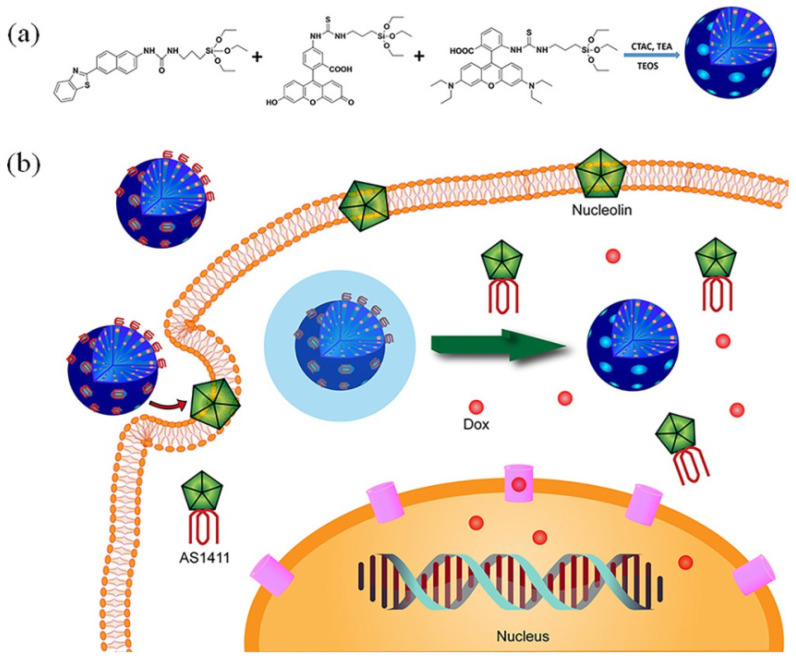
(**a**) Diagram illustrating the synthetic route of MTP–MSNs. (**b**) Design strategy of the MTP–MSNs@DOX@APTAMER theranostic nanosystem and working mechanism for controlled release of therapeutic agents. Reproduced with permission from Ref. [118], Copyright 2021, John Wiley and Sons.

Researchers engaged in biological or medical research usually aim to analyze specific regions of cells or organisms through visual and clear images. To that end, numerous works have determined the biological information of cells, tissues, and even organisms directly by means of a non-invasive optical imaging technique [110,111,112,113]. Many of these studies are based on mesoporous silica functionalized by fluorophores, upconversion nanoparticles, etc. [114]. Typically, many fluorophores can be covalently grafted to a silica matrix via organo-bridged silane [115], while fluorescent inorganic compounds can be easily encapsulated by silica shells [116]. Kim et al. [117] reported a fluorescent probe prepared by covalently immobilizing rhodamine 6G onto mesoporous silica via the silane bridge; the resulting rhodamine 6G silica particle (RSSP) can be used as a fluorescent probe for the detection of Fe (III) and applications in bioimaging. Recently, Wu et al. [118] (Figure 8) prepared tri-dye-doped mesoporous silicon nanoparticles (MTP–MSNs) via the copolycondensation of dye-modified silane during the modified Stöber process. The resulting material can be used for multicolor cellular imaging basing on the FRET-based two-photon fluorescence mechanism. After the loading of DOX, the pores of MTP–MSNs were closed by AS1411 aptamer, which acted as both a pore-blocking agent and a targeting tumor agent. The resulting drug delivery system has a controlled release ability in vivo and was proven to be an ideal material for bioimaging and targeted therapy for specific cancers.

**Figure 9:**
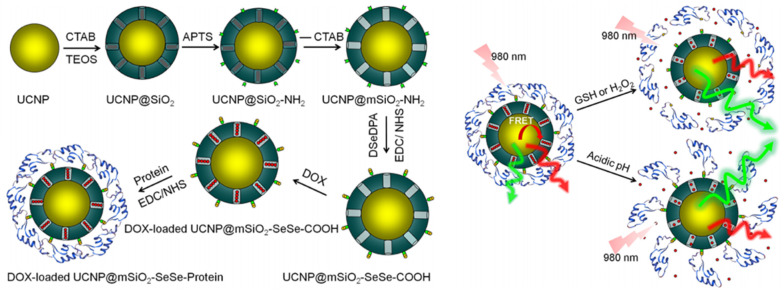
Creation of nanovehicles of protein-gated UCNP@mSiO_2_ via Se−Se linkages to track drug release in real time in response to several stimuli. Reproduced with permission from Ref. [122], Copyright 2021, American Chemical Society.

In general, the integration of sensing imaging abilities with drug delivery is a common topic of research [119,120,121]. For example, Yan et al. [122] (Figure 9) prepared two novel drug delivery nanovehicles with bovine serum albumin- or myoglobin-gated as well as upconverted nanoparticle-embedded mesoporous silica. The resulting UCNP@mSiO_2_ could be used for the controlled release of the encapsulated DOX when activated by tumor microenvironment stimuli such as an acidic pH, glutathione, or H_2_O_2_. In tests on tumor-bearing mice, these drug delivery nanovehicles showed a good inhibitory effect on tumor growth and negligible damage/toxicity to the normal tissues. Meanwhile, the DOX release behavior could be tracked in real time via monitoring the Förster resonance energy transfer between the UCNP cores and DOX. 

**Figure 10:**
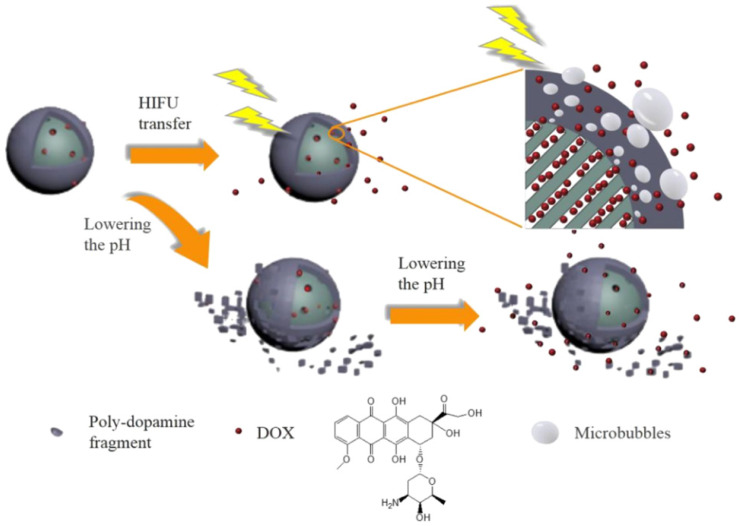
Schematic representation of MSN@DOX−PDA and its pH/HIFU dual-responsive release performances. Reproduced with permission from Ref. [126], Copyright 2021, American Chemical Society.

Recently, stimuli-responsive drug delivery nanovehicles based on mesoporous silica have been widely studied for their ability to control drug release from pores under various stimuli, such as magnetism, ultrasound, light, and so on [123,124,125]. Li et al. [126] (Figure 10) synthesized a novel pH- and ultrasound-responsive DOX-releasing material based on polydopamine-coated mesoporous silica. The as-prepared DOX-loaded nanoparticles displayed a pH-related response induced by the degradation of polydopamine under acidic conditions, which triggers the release of encapsulated DOX from pores upon ultrasound exposure. Furthermore, it was observed that the nanoparticles could be used to inhibit the growth of tumor cells through the controlled release of DOX. Moreover, the nanoparticles possessed a photothermal effect due to NIR adsorption by the polydopamine layer, demonstrating their good potential for chemotherapy in cancer treatment.

**Figure 11:**
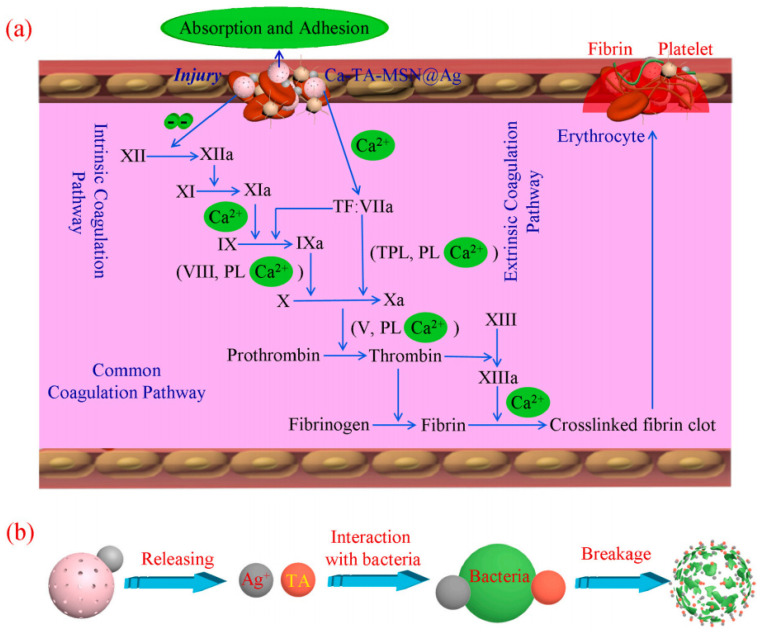
The potential (**a**) hemostatic and (**b**) antibacterial mechanism of Ca-TA-MSN@Ag. Reproduced with permission from Ref. [133], Copyright 2021, Elsevier.

Another important aspect in the biological applications of mesoporous silica relates to the development of novel hemostatic materials [127,128,129,130,131]. The high specific surface area of mesoporous silica enables it to rapidly absorb large amounts of water from the blood, causing a higher concentration of clotting factors and platelets and thus promoting hemostasis. For example, Zhang et al. [132] prepared a large-pore mesoporous silica particle that has a shorter blood clotting time in vitro than Celox™ granules. They further immobilized the mesoporous silica particle onto cotton fibers to form a mesoporous silica–cotton composite, which leads to a shorter blood clotting time and less blood loss than that seen with raw mesoporous silica powder in mouse bleeding models. In addition, mesoporous silica can also be doped with antibacterial drugs to prevent infections. Chen et al. [133] loaded tannic acid (TA), silver nanoparticles, and calcium ions onto mesoporous silica nanoparticles (MSNs) to prepare a novel biomaterial (Ca-TA-MSN@Ag) with the dual functions of hemostatic performance and antibacterial activity (Figure 11). The porosity of MSNs, TA molecules, and calcium ions could lead to a triple synergistic hemostatic effect, and so the time needed for Ca-TA-MSN@Ag to reach hemostasis is shorter than that for pure MSN. Furthermore, Ca-TA-MSN@Ag also showed excellent antibacterial activity due to having loaded Ag nanoparticles and TA molecules.

## 5. Conclusions

Since the discovery of mesoporous silica molecular sieves, their synthesis and application have attracted extensive attention from all over the world. This mini review briefly introduced the history of developmental and biological applications of mesoporous silica molecular sieves. Many significant families of mesoporous silica molecular sieves have been found, including M41s and SBA. The morphology of mesoporous silica has also developed from bulk materials to monodisperse nanoparticles, and some silica even has a hollow structure. Tuning the morphology of mesoporous silica can be achieved via the choice of molecular template or by adjusting the reaction parameters of the solution. By utilizing the well-known Stöber method, monodisperse mesoporous silica microspheres can be obtained. Furthermore, the combination of the seed growth mechanism originating from the Stöber method with a templating method can be used to develop hollow mesoporous silica microspheres with a higher hosting capacity. Additionally, the soft templating methods based on micelle or microemulsion play an important role in the synthesis of hollow mesoporous silica microspheres.

The properties of mesoporous silica molecular sieves, including a high biocompatibility, excellent hosting capability, etc., make them a popular carrier in biological applications. The most important application of mesoporous silicas is as a drug carrier. Generally, uniform spherical and hollow structures improve the drug loading and delivery capacities of mesoporous silica. Meanwhile, the incorporation of targeting, controlled release, and stimuli-responsive functions makes drug-loaded mesoporous silica promising for precision therapy. In addition, the functionalization of drug-loaded mesoporous silica with optical units, such as fluorophores, upconversion nanoparticles, etc., enables us to track the progress of drugs through a non-invasive bio-imaging technique. In the future, novel mesoporous silica-based drug delivery systems that combine functions such as targeting, stimuli responses, controlled release, and optical imaging will be of great significance in the field of disease treatment.

## Figures and Tables

**Table 1:** Information about the origin of several important mesoporous silica families.

Name	Origin of Name	First Report	References
M41S	Mobil Composition of Matter No. 41	Kresge et al./1992	[24,25]
HMS	Hexagonal Mesoporous Silica	Tanev et al./1994	[39]
FSM	Folded Sheets Mesoporous Materials	Inagaki et al./1994	[40]
MSU	Michigan State University	Bagshaw et al./1995	[41]
KIT	Korea AdvancedInstitute of Science and Technology	Ryoo et al./1996	[42]
SBA	Santa Barbara Amorphous	Zhao et al./1998	[33]
FDU	Fudan University	Yu et al./2000	[43]
JLU	JiLin University	Liu et al./2000	[44]
TUD	Technische Universiteit Delft	Jansen et al./2001	[45]
COK	Centrum voor Oppervlaktechemie en Katalyse	Jammaer et al./2008	[46]
HMM	Hiroshima Mesoporous Material	Nandiyanto et al./2009	[47]

## Data Availability

Not acceptable.

## Sample Availability

Samples of the compounds are not available from the authors.
